# Progressive Domain Segregation in Early Embryonic Development and Underlying Correlation to Genetic and Epigenetic Changes

**DOI:** 10.3390/cells10102521

**Published:** 2021-09-23

**Authors:** Hui Quan, Hao Tian, Sirui Liu, Yue Xue, Yu Zhang, Wei Xie, Yi Qin Gao

**Affiliations:** 1 Beijing National Laboratory for Molecular Sciences, College of Chemistry and Molecular Engineering, Peking University, Beijing 100871, China; quanhui@pku.edu.cn (H.Q.); ccme_th@pku.edu.cn (H.T.); liusirui@pku.edu.cn (S.L.); xueyccme@pku.edu.cn (Y.X.); 2 Center for Stem Cell Biology and Regenerative Medicine, MOE Key Laboratory of Bioinformatics, School of Life Sciences, Tsinghua University, Beijing 100871, China; zhangyu-11@mails.tsinghua.edu.cn (Y.Z.); xiewei121@tsinghua.edu.cn (W.X.); 3 THU-PKU Center for Life Sciences, Tsinghua University, Beijing 100871, China; 4 Biomedical Pioneering Innovation Center (BIOPIC), Peking University, Beijing 100871, China; 5 Beijing Advanced Innovation Center for Genomics, Peking University, Beijing 100871, China

**Keywords:** sequence, domain segregation, epigenetic modification, ZGA and implantation

## Abstract

Chromatin undergoes drastic structural organization and epigenetic reprogramming during embryonic development. We present here a consistent view of the chromatin structural change, epigenetic reprogramming, and the corresponding sequence-dependence in both mouse and human embryo development. The two types of domains, identified earlier as forests (CGI-rich domains) and prairies (CGI-poor domains) based on the uneven distribution of CGI in the genome, become spatially segregated during embryonic development, with the exception of zygotic genome activation (ZGA) and implantation, at which point significant domain mixing occurs. Structural segregation largely coincides with DNA methylation and gene expression changes. Genes located in mixed prairie domains show proliferation and ectoderm differentiation-related function in ZGA and implantation, respectively. The chromatin of the ectoderm shows the weakest and the endoderm the strongest domain segregation in germ layers. This chromatin structure difference between different germ layers generally enlarges upon further differentiation. The systematic chromatin structure establishment and its sequence-based segregation strongly suggest the DNA sequence as a possible driving force for the establishment of chromatin 3D structures that profoundly affect the expression profile. Other possible factors correlated with or influencing chromatin structures, including transcription, the germ layers, and the cell cycle, are discussed for an understanding of concerted chromatin structure and epigenetic changes in development.

## 1. Introduction

In mammals, chromatin undergoes drastic organizational and epigenetic reprogramming after fertilization [1,2]. These processes are essential for gene regulation, either globally or specifically, by generating a chromatin environment that is permissive or repressive to gene expression [3]. The chromatin of mouse zygotes and two-cell embryos has obscure high-order structures, existing in markedly relaxed states, which undergo the consolidation of TADs (topologically associating domains, that is, self-interacting domains in the 3D space) and segregation of chromatin compartments through development [4,5]. The TAD structure and compartmentalization are also gradually established following human fertilization [6]. Along with the remodeling of the 3D chromatin architecture, genome-wide epigenetic reprogramming also takes place during embryonic development [2,7,8,9,10,11,12]. The global hypomethylation of the genome occurs, and histone modifications are globally reset, changing from a non-canonical distribution to a canonical one [13,14,15] during early embryonic development, leading to the specification of the germ layers and cell differentiation.

The progression of the mammalian embryo from fertilization to germ-layer formation, concurrent with transcriptional changes and cell fate transitions [3], involves an ordered series of hierarchical lineage determinations that ensures the establishment of a blueprint for the whole animal body. One of the most notable transcriptional changes is the zygotic genome activation (ZGA), during which the embryo changes from a state where there is little transcription to another state in which thousands of genes are transcribed [16]. The ZGA is mechanistically coordinated with changes in the chromatin state and cell cycle [17]. Mammalian ZGA may primarily prepare for differentiation toward the inner cell mass (ICM) and the trophectoderm (TE), which begins at the morula stage, then the TE can further develop into the extraembryonic tissue necessary for embryo implantation and receiving nutrients. After implantation, the ICM then gives rise to three germ layers (ectoderm, mesoderm and endoderm) through gastrulation, generating founder tissues for subsequent somatic development [18,19].

Thanks to recent developments in low-input chromatin analysis technologies, the chromatin structural, epigenetic and transcriptional properties have been roundly explored in the early embryonic development process [20]. Assuming that chromatin structural properties at the mouse pre-implantation stages have been investigated, analysis on the latest Hi-C data describing chromatin structural properties at post-implantation stages in mice and embryonic development in humans ought to be able to provide a fairly complete view of the structural change during development. On the other hand, the relationship between global genome structural changes, epigenetic reprogramming, and the DNA sequence remains largely unknown and, therefore, needs to be investigated further.

In our previous study, we analyzed the DNA sequence dependence in the formation of 3D chromatin structures for different cell types [21]. Based on CpG island (CGI) densities, the genome was divided into alternative forest (high CGI density, F) and prairie (low CGI density, P) domains with average lengths of 1 to 3 million bases (forest and prairie domains are therefore cell type-invariant in one species). CGI forests and prairies effectively separate the linear DNA sequence into domains with distinctly different genetic, epigenetic and structural properties. 78.5% of human genes (and 91.3% of human housekeeping genes) reside in forest domains. Besides, compartments A and B are mainly composed of forest and prairie regions, and the segregation of the two domains tends to intensify during embryonic development, cell differentiation and senescence as a result of sequence-based thermodynamic stabilization. However, the segregation degree of some somatic cells is less than that of ICM, implying that the chromatin structure changes in a non-monotonic way from zygote to differentiated somatic cells. The way that chromatin conformation gradually changes to establish cell identity during development is thus an interesting open question.

In the present study, we conducted an analysis of global chromosome structural changes during early embryonic development. Two specific stages, ZGA and implantation, during which gene activation and lineage specification occur, respectively, were both found to involve the mixing of the two types of genomic domains, with the latter showing a more significant change than the former. The segregation level positively correlates with the proportion of prairie domains residing in compartment B, the larger value of which suggests a more segregated chromatin structure. We also found that, compared to prairie regions constantly residing in compartment B, genes in switchable prairie regions tend to be tissue-specific. The DNA methylation distribution in early embryonic development also correlates with the trend of domain segregation in this process, which indicates that the chromatins of the endoderm and mesoderm are more segregated than that of the ectoderm. Moreover, the domain segregation levels of the earliest fate-committed germ layers correlate with those of the differentiated cells. We further investigated in detail the chromatin structure changes at different scales during ZGA and found a correlation between LAD mixing and domain mixing in ZGA, which was related to the functions of cell proliferation. The detailed functions of genes residing in more mixed domains during implantation were then analyzed, all of which are ectoderm-related. Finally, we present a consistent view of the structural change of chromatin from birth to senescence and discuss possible factors influencing global chromatin structure, such as transcription, germ layers, and cell division.

## 2. Materials and Methods

### 2.1. Overall Segregation Ratio

Based on the Hi-C contact matrix, the inter-domain contact ratio between the same domain types was calculated as:

$$R_{f}^{s}=\frac{\sum_{i,\;j\in F,\;i\neq j}D_{ij}}{\sum_{i\in F,\;j\in A}D_{ij}}\;\mathrm{and}\;R_{p}^{s}=\frac{\sum_{i,\;j\in P,\;i\neq j}D_{ij}}{\sum_{i\in P,\;j\in A}D_{ij}}$$

for forests and prairies, respectively. In the above equations, $D_{ij}$ represents the sum of contacts between the two domains i and j, which is further divided by the product of domain length of i and j. F is the collection for all forest domains, P is the collection for all prairie domains, and A is the union of sets F and P.

The inter-domain contact between different types was calculated similarly as:

$$R_{f}^{d}=\frac{\sum_{i\in F,\;j\in P}D_{ij}}{\sum_{i\in F,\;j\in A}D_{ij}}\;\mathrm{and}\;R_{p}^{d}=\frac{\sum_{i\in P,\;j\in F}D_{ij}}{\sum_{i\in P,\;j\in A}D_{ij}}$$

The overall segregation ratio $OR_{s}$ was then defined as the ratio of inter-domain contacts between the same types and different types:

$$OR_{s}=\frac{R_{f}^{s}}{R_{f}^{d}}\;\mathrm{or}\;OR_{s}=\frac{R_{p}^{s}}{R_{p}^{d}}$$

for forests and prairies, respectively. All Hi-C data in this calculation were normalized by ICE normalization [22].

### 2.2. 3D Chromatin Structure Modeling

Detailed information can be found in our previous work [23]. Briefly, we first coarse-grained a chromosome as a string of beads. The equilibrium distance between two beads was obtained by converting the contact frequency to the spatial distance. A randomly generated initial structure was then used for further structure optimization using MD (Molecular Dynamics) simulations until the RMSD (root-mean-square deviation) of the modeled structure became convergent.

### 2.3. Domain Segregation Ratio Calculation

For each forest/prairie domain i, the domain-segregation ratio $DR_{s}$ was defined as the ratio between its inter-domain contacts with the same types and its inter-domain contacts with different types:

DRsi∈F=∑j∈F,j≠iDij∑j∈PDij and DRsi∈P=∑j∈P,j≠iDij∑j∈FDij

In the above equations, $D_{ij}$ represents the sum of contacts between the two domains i and j, F is the collection of all forest domains, and P is the collection of all prairie domains.

### 2.4. Distance-Dependent Segregation Ratio Calculation

The segregation ratio at each distance d was calculated as the ratio of contacts with that distance apart between the same types and different types:

$$R_{s}^{f}\left(d\right)=\frac{\sum_{i\in F,j=i\pm d\in F}C_{ij}}{\sum_{i\in F,j=i\pm d\in P}C_{ij}}\;\mathrm{and}\;R_{s}^{p}\left(d\right)=\frac{\sum_{i\in P,j=i\pm d\in P}C_{ij}}{\sum_{i\in P,j=i\pm d\in F}C_{ij}}$$

for forests and prairies, respectively. In the above equations, $C_{ij}$ is the normalized Hi-C contact probability between bins i and j, F is the collection for all forest domains, and P is the collection for all prairie domains.

### 2.5. Open-Sea Methylation Difference Index (MDI) between Forest and Prairie

We quantified the methylation difference between adjacent forests and prairies by:

$$MDI_{i}=\left(q_{i}-\frac{q_{i-1}+q_{i+1}}{2}\right)/\left(\frac{q_{i-1}+q_{i}+q_{i+1}}{3}\right)$$

where $q_{i}$,$\;q_{i-1}$ and $q_{i+1}$ are the average open-sea methylation levels for the i th domain and its two flanking domains.

### 2.6. Overall Relative Segregation Ratios

Overall relative segregation ratios ($R_{or}$) between one tissue and another were identified as:

$$R_{or}=ln\left(\frac{N_{ij}^{o}}{N_{ji}^{o}}\right)$$

where $N_{ij}^{o}$ is the number of domains possessing higher $DR_{s}$ value in tissue i, compared to tissue j. A positive value of $R_{or}$ indicates that the chromatin of the former tissue is more segregated than that of the latter. Thus, the parameter $R_{or}$ is used to generally reflect domain-segregation behavior differences between two tissues.

### 2.7. Significantly More Segregated or More Mixed Domains

To identify forest or prairie domains that are significantly more (or less) segregated in one tissue when compared to another, we first identified the threshold values to distinguish more (or less) segregated domains at certain stages. We calculated the logarithm ratio of $DR_{s}$ values between the two ICM replicates from the same laboratory. Significantly more strongly segregated domains are taken as those where the variation of $DR_{s}$ values are in the top 2.5-percent tier of all domains, and more mixed domains in the bottom 2.5 percent. The corresponding threshold values of $DR_{s}$ variation $T_{DR_{s}}^{log}$ for segregation, and mix are then 0.1469 and −0.1469, respectively. 

We calculated the logarithm ratio of $DR_{s}$ between early and late 2-cell embryos for ZGA, ICM and E6.5 epiblast for implantation, respectively. Significantly more strongly segregated domains were identified as those with a logarithm ratio of $DR_{s}$ that is higher than 0.1469, and significantly more mixed domains were defined as those with a logarithm ratio of $DR_{s}$ that is lower than −0.1469. For convenience, we denoted more strongly segregated and more mixed domains in forest and prairie as $F_{seg}$, $P_{seg}$, $F_{mix}$, and $P_{mix}$, respectively.

### 2.8. Compartment Index Calculation

To quantify the degree of compartmentalization, we defined a compartment index (C-index) $I_{i}$ for 200-kb bin i as the logarithm ratio of the average contact between this bin and all A compartments, over that between this bin and all B compartments:

$$I_{i}=ln\frac{\left(\sum_{j}C_{ij}\delta_{j}\right)/N_{A}}{\left(\sum_{j}C_{ij}(1-\delta_{j})\right)/N_{B}}\;\delta_{j}=\{\begin{array}{c} 1,\;\mathrm{if}\;\mathrm{bin}\;j\;\mathrm{is}\;\mathrm{in}\;\mathrm{compartment}\;A\\ 0,\;\mathrm{if}\;\mathrm{bin}\;j\;\mathrm{is}\;\mathrm{in}\;\mathrm{compartment}\;B \end{array}$$

where $C_{ij}$ is the distance-normalized Hi-C contact probability (normalized by dividing each contact by the average contact probability at that genomic distance) between bins i and j. $N_{A}$ and $N_{B}$ are the total number of bins belonging to compartments A and B, respectively. For each region, a higher value of $I_{i}$ indicates a more compartment A-like environment, and a lower one, a compartment B-like environment.

### 2.9. Gene Function Analysis

We analyzed the functional enrichment of genes located in various selected regions using ClusterProfiler and DAVID (https://david.ncifcrf.gov, accessed on 16 March 2021), which yielded similar results. The results were demonstrated via GO terms with *p*-values. The functional annotation clustering was analyzed using DAVID, and the results were shown via GO terms with enrichment scores.

### 2.10. Identification of Lineage-Specific Genes

To identify lineage-specific genes, we used a Shannon-entropy-based method [24]. Genes with an entropy score of less than 1.7 were selected as candidates for stage-specific genes. Among them, we selected E6.5 epiblast-specific genes that satisfied the following conditions: the gene is highly expressed in E6.5 epiblast (FPKM ≥ 1); its relative expression is higher than 1/7; and its expression level in epiblast is higher than that in ICM. These genes were then reported in the final lineage-specific gene lists.

### 2.11. Comparison of Segregation Extent between SINE and CpG Density

For each 40 kb bin (the resolution corresponds to Hi-C matrix resolution), we calculated its CpG and SINE density. Bins where the CpG (SINE) density was larger than the median value of CpG (SINE) density were regarded as high-CpG (SINE) bins, and the remaining bins were low-CpG (SINE) bins. For the two groups of bins, such as hChS and hClS, the segregation extent was calculated as the average contact probability between hChS and hChS, divided by hChS and hClS, and the average contact probability between hClS and hClS, divided by hChS and hClS, respectively.

## 3. Results

### 3.1. Domain Segregation in Early Embryonic Development

Previous studies on chromatin structures during early embryonic development were mainly about changes to structural elements, such as compartments and TADs [4,5]. Here we present a structural analysis based on two types of genomic domains, to show domain segregation behaviors in different scales. We analyzed chromatin structural changes by calculating the overall segregation ratio $OR_{s}$ (one parameter used to globally reflect chromatin segregation behavior), the domain segregation ratio $DR_{s}$ (which was used to reflect segregation behavior of individual forest/prairie domains), the segregation ratio regarding genomic distances $R_{s}\left(d\right)$ and the F–F (forest–forest)/F–P (forest–prairie)/P–P (prairie–prairie) spatial interaction ratio at each stage.

In our previous study [21], based on the division of the mammalian genomes into forest and prairie domains, $OR_{s}$ was defined as the inter-domain contact ratio between domains of the same types and different types, regarding each domain as one unit (see “Methods”, for instance, $OR_{s}$ for forest is calculated as forest–forest interactions divided by forest–prairie interactions). A higher $OR_{s}$ for a sample indicates a stronger segregation. Here, we calculated $OR_{s}$ using Hi-C data [4,6,20] of 21 mouse cells and 5 human cells (see Additional File 2: Data Sources), which allowed us to investigate the chromatin structure changes following early embryonic development. The calculated $OR_{s}$ for each stage of mouse and human embryo development is given in 1A and 1B, respectively. We also constructed the modeled 3D chromatin structures following our previous work [23] to show visually the degree of segregation at each stage (see “Methods”).

**Figure 1:**
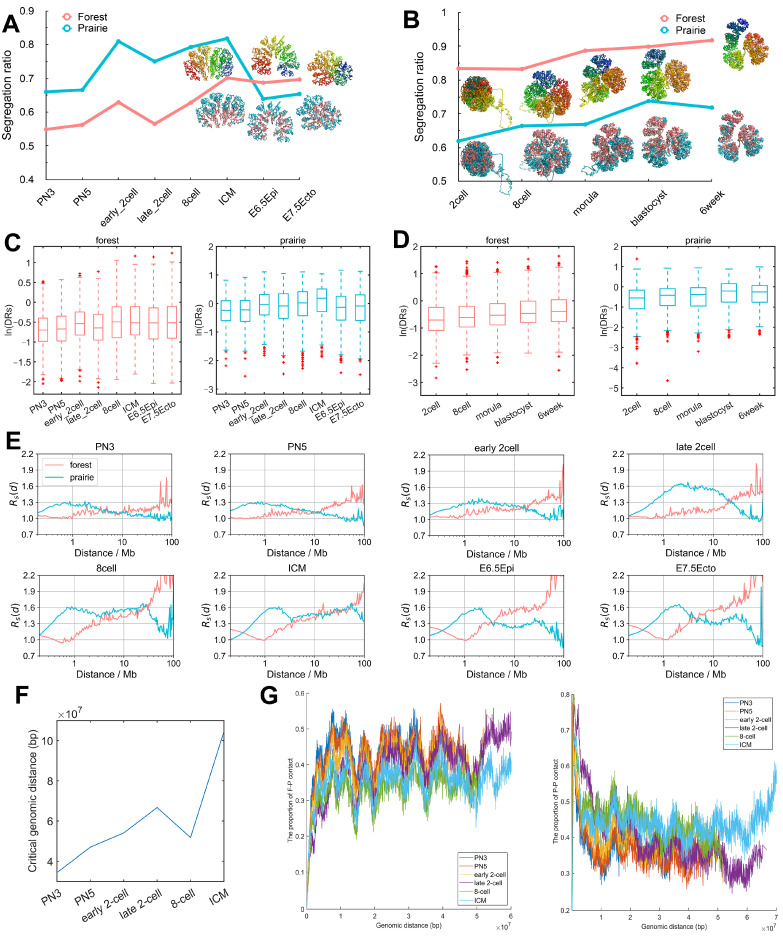
Domain segregation in early embryonic development. (**A**,**B**) The chromatin overall segregation ratio $OR_{s}$ of forest and prairie domains at each stage in mouse (**A**) and human (**B**) embryonic development. Modeled chromatin structures are also shown at corresponding stages. The upper structures are colored by the sequence from blue to red, and the lower structures are colored by forest/prairie domains. (**C**,**D**) The domain segregation ratio $DR_{s}$ of forest and prairie domains at each stage in mouse (**C**) and human (**D**) embryonic development. (**E**) The distance-dependent segregation ratio $R_{s}\left(d\right)$ of forest and prairie at each stage in mouse embryonic development. (**F**) The change of critical genomic distance during mouse embryonic development. (**G**) The proportions of F–P and P–P spatial interactions within the top 10% contact probabilities under one certain genomic distance, at each stage in mouse embryonic development.

As seen from Figure 1A, $OR_{s}$ increases during the development of early mouse embryonic cells, except for two dips. Such a trend suggests the increased segregation of forest and prairie domains from each other, in line with our previous observation that more inter-compartment interactions occur at early stages than late stages [4]. The two dips observed along the $OR_{s}$ curve correspond to the early to late 2-cell, and ICM to E6.5 epiblast transitions, respectively. The decrease during the former transition is seen in both alleles (Figure S1A). For the latter transition, the modeled chromatin structure clearly changes from a domain-separated state to a forest–prairie (F–P) mixed state (Figure 1A). Interestingly, these two processes (that is, early to late 2-cell, and ICM to E6.5 epiblast) exactly coincide with ZGA [25] and implantation [26], respectively. The domain-level F–P chromatin mixing suggested by the decrease in $OR_{s}$ is possibly related to LAD (lamina-associated domains) mixing at the ZGA stage, as well as germ layer differentiation following implantation, which is analyzed below.

The overall chromatin segregation follows a similar trend in human embryonic development (Figure 1B). The $OR_{s}$ for prairies generally increases except for the blastocyst-to-6-week transition, in accordance with the observed trend of global segregation and domain-level mixing after the ICM stage in the mouse sample. These changes of domain segregation are also reflected by the 3D structures reconstructed via Hi-C data (Figure 1B). Again, these results suggest that forest and prairie domains tend to segregate from each other before implantation, and domain mixing is observed at the post-implantation stage, showing an increased interaction of the prairie with forest domains. 

Besides the overall structure parameter $OR_{s}$, we also calculated the domain segregation ratio $DR_{s}$ on each forest/prairie domain during embryonic development (see “Methods”; a higher value indicates that the corresponding domain is more segregated), and the variation trend is similar to $OR_{s}$ (Figure 1C (mouse) and Figure 1D (human)). Interestingly, for the mouse embryo, the change in $DR_{s}$ is more significant for the ICM to E6.5 epiblast transition $P=3.7\times 10^{-96}$ by Welch’s unequal variance test) than the early to late 2-cell transition $P=1.9\times 10^{-16}$ by Welch’s unequal variance test) for prairie domains, while for forest domains the former one is less pronounced ($P:2.4\times 10^{-5}\;\mathrm{VS}\;2.1\times 10^{-54}$, Welch’s unequal variance test).

Furthermore, to investigate chromatin segregation at different genomic distances, we calculated the segregation ratio as a function of genomic distance $R_{s}\left(d\right)$ (Figure 1E, Figures S1B–D and S2, see “Methods”). It can be seen from Figure 1E that in early mouse embryo development, prairies are characterized by elevated segregation at genomic distances of several million bases (Mb). The segregation becomes more pronounced at long distances (>1 Mb) after the 2-cell stage for both forests and prairies, as is again consistent with an enhancement in domain segregation. Besides, Figure S1B shows a slight decrease of $R_{s}\left(d\right)$ for forests at the early to late 2-cell stage and a conspicuous decrease of $R_{s}\left(d\right)$ for prairies at the ICM to E6.5 epiblast stage for mouse embryos; the underlying biological significances behind these phenomena will be discussed below. Enhanced segregation at a several-Mb scale also occurs during human embryonic development (Figure S1C), and an obvious drop in $R_{s}\left(d\right)$ for prairies at the blastocyst-to-6-week transition in human embryos was also observed (Figure S1D). 

Finally, the top 10% of contact probabilities under the genomic distance d (that is, the top 10% of elements of one diagonal of the Hi-C matrix), $TC_{10}\left(d\right)$, were extracted, within which the proportions of F–F, F–P and P–P interactions were calculated (notably, the premise of such a calculation is that all elements in the $TC_{10}\left(d\right)$ should be non-zero, otherwise the proportions of F–F, F–P and P–P are assigned zero). The genomic distance, after which the proportions of F–F/F–P/P–P remain at zero, is named the critical genomic distance. During the development of mouse embryos, the critical genomic distance gradually increases (Figure 1F), showing the gradual establishment of long-range chromatin contact. At the same time, the F-P ratio descends, while the P–P ratio increases from the PN3 to ICM stages, again hinting that forest and prairie become more spatially segregated, along with the development (Figure 1G). These phenomena were also observed in human embryo development (Figure S1E).

### 3.2. Compartment Changes Related to Domain Segregation

**Figure 2:**
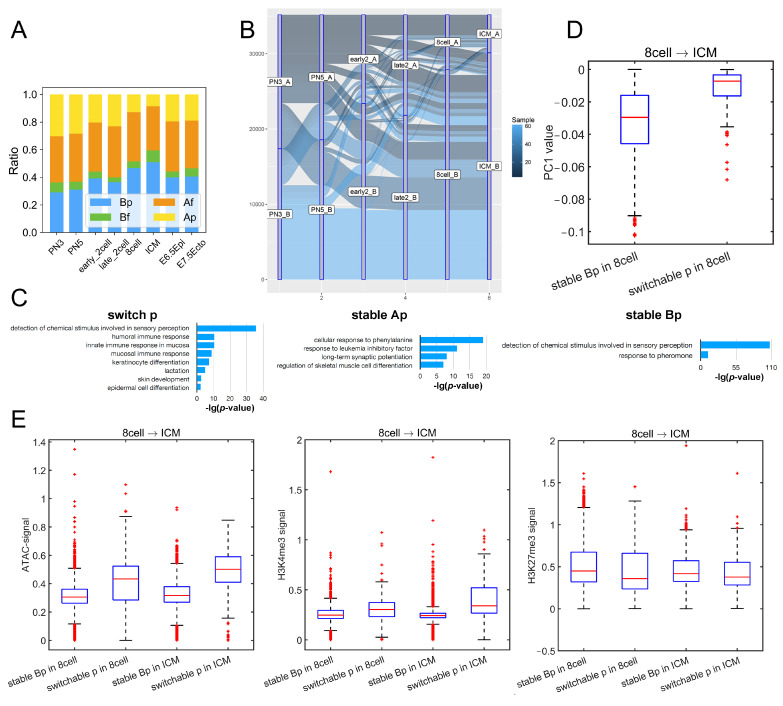
Compartment changes related to domain segregation. (**A**) The proportions of four sequence components, Af, Bf, Ap, Bp (calculated according to the positioning of forests and prairies in compartments **A** and **B**), for each stage in mouse embryonic development. (**B**) The dynamic partition of prairie into compartments **A** and **B** in mouse embryonic development. (**C**) Function annotation clustering of genes located in switch p, stable Ap and stable Bp regions, respectively. (**D**) The PC1 values of stable Bp (i.e., prairie regions constantly remaining in compartment B in both 8-cell and ICM) and switchable p (i.e., prairie regions located in compartment B in 8-cell while switching to compartment A in ICM) regions in 8-cell. (**E**) From 8-cell to ICM, the ATAC-seq, H3K4me3 and H3K27me3 signals of stable Bp and switchable p regions (see notes for Figure 2**D**) in 8-cell and ICM.

By means of the Hi-C measurement, chromatin can be partitioned into two spatial compartments (Figure 2A,B) [27]. It was also found that forest domains reside mainly in compartment A and prairie domains mainly in compartment B in different cells, manifesting that DNA with similar sequence properties tends to spatially interact [21]. Based on these findings, we calculated the proportion for the two sequential domains (forests and prairies) as distributed in the two spatial compartments A and B during development.

The corresponding four types of DNA sequences are named as Af, Bf, Ap, and Bp, respectively, with the first letter denoting the compartments and the second, the forest (f) or prairie (p) domain. One can see from Figure 2A that, during mouse development, the proportion of prairies in compartment B (Bp) changes in the same trend as the overall segregation ratio, which increases from the PN3 to ICM stage, except for the early to late 2-cell stage, then decreases from ICM to E6.5 epiblast, and slightly increases when the E6.5 epiblast develops into the E7.5 ectoderm. The ratio of Ap changes in a direction opposite to that of Bp. A similar phenomenon was also observed in human embryos, where the changes of $OR_{s}$ are in accordance with the size of Bp (Figure S3A). A recent study also found that the attractions between heterochromatic regions are crucial for the compartmentalization and domain segregation of the active and inactive genome [28]. Similarly, we found here that, during embryo development, the aggregation behavior of prairie domains (low CpG density region) strengthens, and more and more prairie regions belong to compartment B.

Since the genome compartmentalization is weak at the early stages of development [4], for the robustness of the compartment partition, we calculated the distribution of the compartment index (C-index, a parameter to quantify the degree of compartmentalization, a larger value of which corresponds to a more compartment-A-like environment; see “Methods”) at each stage of mouse embryo development for compartments A and B, respectively. The gradually increased discrepancy of the C-index between compartment sA and B supports the global structure segregation from zygote to ICM (Figure S3B). Then, we identified compartment-A regions with a positive C-index as strict compartment A (sA), B regions with a negative C-index as strict compartment B (sB), and calculated the length of the genome located in sAf (forest regions in sA), sAp, sBf and sBp, respectively. The result (Figure S3C) showed a trend similar to Figure 2A; that is, the proportion of prairies in strict compartment B (sBp) changes in the same trend as the chromatin segregation level, which rules out the possibility that the growth of Bp is an artifact due to compartment definition.

In comparison with stable Bp regions, the prairie domains that switch the compartment type are particularly interesting, as they may be regarded as mediators of nuclear architecture establishment during development. The analysis of compartment transformation during mouse preimplantation development shows that 7.7% of prairies belong to compartment A (stable Ap), 26.3% of prairies always reside in compartment B (stable Bp), and the remaining 66.0% switch compartment type at least once (switchable p) (Figure 2B). Genes in the stable Ap and switchable p regions are enriched in immune- and ectoderm-related functions, while those in the stable Bp regions are not (Figure 2C). Previous analyses showed that forest and prairie domains tend to spatially segregate (and mainly correspond to compartment A and B, respectively), but to a different extent in different cells [21]. Moreover, forest–prairie spatial intermingling is cell-type specific, which is thought to be associated with prairie tissue-specific gene activation and the establishment of cell identity. Therefore, compartment B (heterochromatin) provides a silent environment for prairie genes, which wait to be activated through spatial interactions with forest/compartment A in the following differentiation stages (as seen in their spatial contact and expression properties in differentiated cells [29]). The function of genes in the stable Ap and switchable p regions thus supports that the gene expression in prairie domains plays an important role in cell fate determination. Indeed, our earlier analysis shows that prairie genes in compartment A are highly tissue-specific and, by examining the functions of the related genes, one can deduce the tissue type of the associated sample [21]. Besides, compared to forest genes, the expression of genes in prairie domains is more likely to be highly correlated with the compartment environment in which they reside [29].

Next, to further investigate the differences between switchable prairie and stable prairie regions, we compared the chromatin features of these two kinds of regions between two adjacent stages. The cells we analyzed here include PN5, early 2-cell, late 2-cell, 8-cell and ICM, due to the availability of mouse Hi-C data. For every two adjacent stages (i.e., PN5 vs early 2-cell, early 2-cell vs late 2-cell, late 2-cell vs 8-cell, 8-cell vs ICM), we identified stable Bp regions and switchable p regions (p regions located in compartment B in the earlier stage, then switched to compartment A in the later stage). Although these two kinds of prairie regions are both located in compartment B in the cell of the earlier stage, the PC1 value of switchable p regions was significantly more positive than stable Bp regions (Figure 2D and Figure S3D); accordingly, switchable p regions are significantly closer to A–B compartment boundaries (Figure S3E), indicating that Bp regions near the A–B boundary are more likely to switch. The analyses on ATAC-seq signal, H3K4me3 and H3K27me3 also support this observation (Figure 2E). For example, from 8-cell to ICM, the ATAC-seq and H3K4me3 signals of switchable p regions are significantly higher than stable Bp regions in both the 8-cell and ICM, while the H3K27me3 signal of switchable p regions is weaker than stable Bp regions.

### 3.3. The Association between Domain Segregation and DNA Methylation

**Figure 3:**
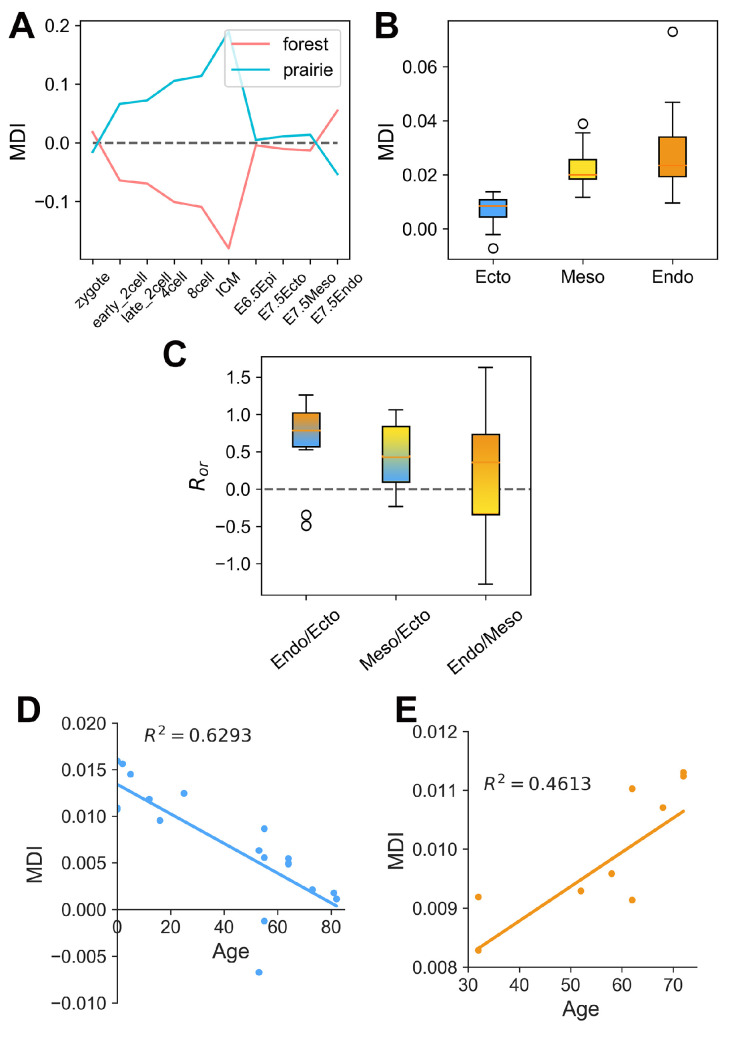
The association between domain segregation and DNA methylation. (**A**) The average forest–prairie open-sea methylation level difference (MDI) at different stages during mouse embryonic development. (**B**) The box plots of the average MDI for human differentiated cells originating from endoderm, mesoderm and ectoderm, respectively, for forest domains. (**C**) The box plots of the overall relative segregation ratio $R_{or}\;$(see “Methods”) of one tissue over another for forests in human embryos. The three categories are tissues derived from endoderm and mesoderm over those derived from ectoderm, and endoderm over mesoderm. The parameter $R_{or}$ was used to evaluate the differences in domain segregation behaviors between two tissues. For example, as for tissues 1 and 2, $N_{12}$ and $N_{21}$ represent the number of domains showing a higher $DR_{s}$ in tissues 1 and 2, respectively. The overall relative segregation ratio for tissues 1 and 2 is then defined as $\ln\left(N_{12}\right)-\ln\left(N_{21}\right)$, a positive value of which thus indicates that the chromatin structure of tissue 1 is more segregated, compared to tissue 2. (**D**,**E**) The scatter-plots of age and methylation difference for brain cells (**D**) and mature neutrophil cells (**E**) for forest domains in human embryos.

In the earlier study, we showed that differences in the methylation levels between forests and prairies correlate well with chromatin spatial packing [21]. In the following, we analyzed methylation data for early mouse embryonic cells obtained by four different research groups (see Additional File 2: Data Sources). These data all show that the open-sea (defined as the genomic regions excluding CGIs, CGI shores and CGI shelves [30]) methylation differences between forest and prairie domains in different cell types correlate well to their corresponding chromatin structural segregation behaviors (Figure 3A and Figure S4A–C). During mouse embryonic development, the absolute value of MDI (F–P open-sea methylation difference index, see “Methods”, where a domain possessing a positive (negative) value indicates the methylation level of this domains is generally higher (lower) than its two flanking domains; therefore, a higher absolute value indicates the larger methylation level difference) increases from 2-cell to ICM stages, decreases at the ICM to E6.5 epiblast stage, and increases again in the further development to the E7.5 stage (especially for the E7.5 endoderm stage, Figure 3A). The variation of DNA methylation difference resembles that of the chromatin structural segregation. The correspondence between methylation difference and segregation degree during mouse embryonic development further supports a connection between this epigenetic mark and the chromatin structure. In fact, such a correlation might have a simple explanation: since forests contribute dominantly to the more accessible chromatin regions, they are presumably more prone to both DNA demethylation and methylation than prairies.

Following such an observation, the extent of domain segregation of three germ layers can be inferred from the DNA methylation difference between the forest and prairie domains. As can be seen from Figure 3A, the calculated absolute value of MDI for the endoderm is greater than that for the mesoderm (the corresponding value of the ectoderm is slightly less than that of the mesoderm). This result suggests that the forest–prairie domains of the endoderm are more segregated than those of the mesoderm and ectoderm, which is indeed in accordance with the analysis on mouse Hi-C data (unpublished results). Interestingly, the domain segregation levels of the different tissues can be seen to reflect the germ layers from which they originate, as shown below from the analyses of both DNA methylation and the structural data.

It was found, based on the Hi-C data, that ectoderm-derived cortex chromatin is less segregated than that of the endoderm-derived liver [21]. Since the above analysis suggests chromatin in the ectoderm to be less segregated than endoderm (Figure 3A), we decided to examine whether chromatin structures of differentiated tissues also show such a trend, therefore exhibiting a germ-layer dependence. We analyzed the average methylation difference levels of 138 mouse differentiated cells and 45 human differentiated cells (38, 34, 66 cells originate from endoderm, mesoderm and ectoderm, respectively, for mouse embryos and 13, 19, 13 for human embryos, see Additional File 2: Data Sources) [31,32,33]. As shown in Figure 3B, the absolute values of MDI for forest domains for human ectoderm-derived tissues are significantly smaller than those for mesoderm- ($P=4.938\times 10^{-7}$ by two-sample *t*-test) and endoderm-derived $P=5.696\times 10^{-4}$ by two-sample *t*-test) tissues, in the same as the segregation degree of the three embryonic germ layers. The data on the prairie domains of human tissues show the same trend, as do those on both the forest and prairie domains of mouse tissues (Figure S4D–F). To further validate this finding, we analyzed the Hi-C data of 14 human tissues [34] (see Additional File 2: Data Sources) and compared the segregation ratio of these tissues, derived from different germ layers. To quantify the difference between two tissues, we defined and calculated the overall relative segregated ratios $R_{or}$) of one tissue over another, a positive value of which represents that chromatin in the former is more segregated than that in the latter (see “Methods”). For human tissues derived from the endoderm and mesoderm, their overall relative segregated ratios over those originating from the ectoderm (cortex and hippocampus) are generally positive (Figure 3C), indicating that the chromatin of the former indeed tends to be more segregated than that of the latter. Similarly, the overall relative segregation ratios of tissues derived from the endoderm over those derived from mesoderm also tend to be positive (Figure 3C and Figure S4G). Together, these results are all consistent with the notion that the segregation level of a differentiated cell shows the corresponding germ layer signature, that is, the order of segregation degrees for the three germ layers is in accordance with that of differentiated tissues.

More interestingly, we found that the absolute values of MDI for the brain decrease with aging, and the trend is opposite for mature neutrophil cells in humans, as shown in Figure 3D,E. Earlier studies have found that DNA hypomethylation, which is more likely to occur in prairie domains, correlates with the cell cycles. Partially methylated domains (PMDs) in tumor cells are mainly composed of prairies [35], and PMD hypomethylation increases with age, which appears to track the accumulation of cell divisions [36]. Xuan Ming et al. also found that solo-WCGW sites display aging- and cancer-associated hypomethylation, which exhibits low maintenance efficiency during the cell cycle [37]. For chromatin structural changes, we compared the segregation ratio in the G1 stage to that in the late S~G2 stage, and found that forests and prairies tend to become more separated in the late S~G2 stage [35]. The enhanced segregation supports the hypothesis that mitosis is conducive to a more segregated chromatin structure. Therefore, we speculate that the different methylation patterns associated with aging among different tissues may reflect their different cell division patterns: the ectoderm-originating brain cells hardly divide, while liver cells constantly undergo cell cycles. The observed MDI differences between these cells are consistent with their different dividing patterns in life. Such consistency makes an understanding of the mechanistic connection between methylation and cell-division patterns highly desirable.

### 3.4. ZGA and Associated 3D Genome Architecture Change

**Figure 4:**
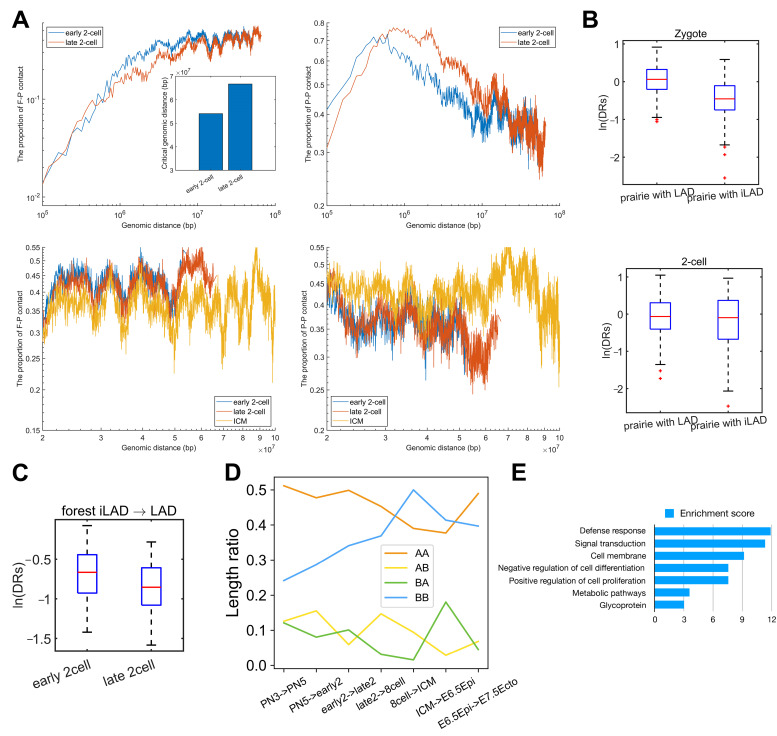
ZGA and associated 3D genome architecture change. (**A**) The upper two figures represent the proportion of F–P and P–P spatial interactions within the top 10% contact probabilities under one certain genomic distance, for normal mouse early 2-cell, late 2-cell. The lower two figures were the amplifications of the partial regions of two upper figures and ICM was also included for comparison. The inner plot represents the critical genomic distance of early 2-cell and late 2-cell. (**B**) The domain segregation ratio ($DR_{s}$) of prairie domains that are/are not in LAD regions in mouse embryos. (**C**) The domain segregation ratio of forest domains switching from iLAD to LAD during mouse ZGA, in early 2-cell and late 2-cell. (**D**) The proportion of four components (AA: A→A, AB: A→B, BA: B→A, BB: B→B) in each transition of mouse embryos (e.g., from early 2-cell to late 2-cell). The length ratios of, e.g., AA for PN3 -> PN5 indicates the ratio between the genome length of stable A regions (between PN3 and PN5) and the entire genome length. (**E**) Function annotation clustering of prairie genes switching from B to A during mouse ZGA.

To further investigate how transcription is associated with chromatin structure, we analyzed the chromatin structure changes from mouse early 2-cell to late 2-cell at different genomic distance scales, since ZGA typically occurs at this period. At small genomic distances (<500 kb), the F–P ratio for the late 2-cell is larger than that for early 2-cell, while the P–P ratio is smaller for the former (Figure 4A, upper two figures). Our previous work has revealed that prairie–forest intermingling was associated with prairie gene activation [21]; therefore, we speculate that the increase of F–P ratio may be associated with ZGA. At large distances (500 kb~20 Mb), the P–P ratio (F–P ratio) of the late 2-cell was larger (smaller) than the early 2-cell (Figure 4A, upper two figures), indicating that at such a scale, the forest–prairie separation is enhanced, in accordance with the increase of compartmentalization degree [4] (given that compartment A and B are mainly composed of forest and prairie regions, respectively). Furthermore, compared to the early 2-cell, the chromatin of a late 2-cell establishes more spatial interactions over longer distances (>50 Mb, Figure 4A, down two figures). However, the compartmentalization is weak at this scale (compared to the F–P/P–P ratio of ICM in the same range (i.e., >50 Mb) and the late 2-cell itself in a small range (i.e., <50 Mb), Figure 4A, down two figures). 

LAD domains, which resemble compartment B, are established quickly after fertilization. More than 75% of LAD domains are prairies across preimplantation development in mouse embryos, and the domain segregation ratio ($DR_{s}$) of prairie domains overlapping with LAD is significantly larger than those that are not (Figure 4B, $P=2.9\times 10^{-34}$ and 0.01 for mouse zygote and 2-cell, respectively, Welch’s unequal variance test). We further analyzed the correlation between LAD mixing and prairie/forest mixing at the ZGA stage in mouse embryos. LAD/iLAD (inter-LAD) regions in the zygote and 2-cell stages were obtained from previous studies [38]. We then identified forest/prairie domains that become significantly more intermingled or segregated (i.e., $F_{mix}$$F_{seg}$/$P_{mix}$/$P_{seg}$, see “Methods”) during mouse ZGA. The number of forest domains switching from an iLAD to LAD state is 139, half of which (70 domains) belong to $F_{mix}$. In contrast, the proportion of $F_{mix}$ domains within forest domains remaining in the iLAD state is only 24.8% ($P=1\times 10^{-7}$ by Fisher’s exact test). Accordingly, forests that change from iLAD to LAD regions show a significant decrease in domain segregation ratios (Figure 4C, $P=6.7\times 10^{-5}$ by two-sample t-test). Such a result indicates that the forests changing from iLAD to LAD gain contacts with prairies, and a correlation does exist between LAD-mixing and forest-mixing during ZGA. 

We next examined how a compartment switch is associated with ZGA. Firstly, we examined the compartment change during mouse embryonic development. The overall length of genomic domains changing from compartment A to B is shorter than those switching from compartment B to A in ZGA (Figure 4D). Such a phenomenon was also observed in implantation (from ICM to E6.5Epi). In addition, the prairie genes that move from compartment B to A in ZGA were found to be rich in the functional annotation of “defense response”, “signal transduction”, “cell membrane”, “negative regulation of cell differentiation” and “positive regulation of cell proliferation,” identified using DAVID [39,40] (see “Methods”, Figure 4E). These results indicate that the domain mixing during ZGA is heavily related to the functions of cell proliferation.

### 3.5. Implantation-Related Domain Mixing in Differentiation

**Figure 5:**
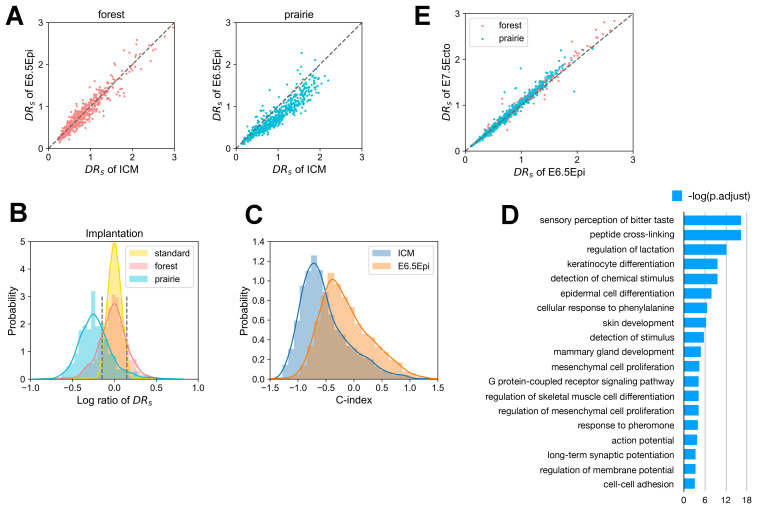
Implantation-related domain mixing in differentiation. (**A**) The scatter diagrams of the domain segregation ratio $DR_{s}$ for forests and prairies during mouse implantation. (**B**) The distribution of the logarithm ratio of $DR_{s}$ between mouse E6.5Epi and ICM for forests and prairies. The plot, the legend of which is “standard”, represents the logarithm ratio of $DR_{s}$ between two ICM replicates from the same lab. Significantly more strongly segregated domains are taken, as those whose logarithm ratio of $DR_{s}$ values exceed the 97.5th percentile of the “standard” distribution; accordingly, domains where the logarithm ratio of $DR_{s}$ is smaller than the 2.5th percentile of the “standard” distribution are regarded as significantly more mixed domains. The corresponding threshold values are 0.1469 and –0.1469, respectively, which was labeled using two black dotted lines. (**C**) The change of compartment index (C-index) for less-segregated prairies, $P_{mix}$, from the mouse ICM to E6.5 epiblast stage. (**D**) Functional assignment of genes in the $P_{mix}$ during mouse implantation. (**E**) The scatter plot of $DR_{s}$ for forests and prairies between the mouse E6.5 epiblast and E7.5 ectoderm.

Since it was shown that the forest–prairie inter-domain interaction coincides with tissue-specific gene activation in differentiation [21], one wonders how the achievements of tissue-specific functions are reflected by domain mixing in implantation. We again calculated the domain segregation ratio $DR_{s}$ for each forest/prairie domain at related mouse embryonic developmental stages (i.e., ICM and E6.5Epi), and observed that $DR_{s}$ decreases for 89.0% of prairie domains at implantation (Figure 5A). To be more specific, we identified those DNA domains that undergo significant changes of segregation, and denoted more strongly segregated and more mixed domains in forest and prairie as $F_{seg}$, $P_{seg}$, $F_{mix}$, and $P_{mix}$, respectively (see “Methods”). In the implantation stage, 71.0% of prairie domains belong to $P_{mix}$, while 21.3% of forest domains become more mixed (Figure 5B). 

As for $P_{mix}$ regions following mouse implantation, we first examined their structural changes by calculating the compartment index (C-index) changes, a larger value of which corresponds to a more compartment A-like environment (see “Methods”). Along with the ICM to E6.5 epiblast transition, 86.9% of $P_{mix}$ domains have an increase in the C-index (entering a more compartment A-like environment), implying that genes in these regions move to a more active environment (Figure 5C). Next, we analyzed the functional enrichment for genes located in the $P_{mix}$ domains using ClusterProfiler [41] and DAVID [39,40] (see “Methods”). The associated $P_{mix}$ genes are characterized by “sensory perception of taste”, “regulation of lactation”, “keratinocyte differentiation”, “epidermal cell differentiation”, “epidermis development”, “skin development”, “mammary gland development” and “long-term synaptic potentiation” (Figure 5D). Interestingly, these terms are related to mammary glands, the epidermis and nervous system, all of which are differentiated from the ectoderm [42]. In fact, chromatin structures of E6.5 epiblast and E7.5 ectoderm are also similar, as seen by their similar $DR_{s}$ values (Figure 5E). In addition, during the mouse implantation process, the expression level of 309 prairie genes increased, while 162 prairie genes showed a decreased expression level. Here, we further found that prairie domains harboring genes with elevated expression levels tend to become more forest–prairie-mixed from ICM to E6.5Epi, compared to genes with lowered expression (Figure S5A, $P=4.163\times 10^{-4}$ by two-sample *t*-test), as is consistent with our previous finding on the vital role of forest–prairie spatial interactions in prairie gene activation [21]. Moreover, we found that $P_{mix}$ genes with increased expression tend to be enriched in neuro-related functions (Figure S5B), indicating that the neuroectoderm may differentiate ahead of the epidermal ectoderm. Finally, to confirm the roles of domain segregation changes on biological functions, we analyzed the structural changes of domains containing lineage-specific genes. We identified 552 lineage-specific genes for the E6.5 epiblast stage (see “Methods”, i.e., genes specifically and highly expressed in the E6.5 epiblast stage), among which 474 genes locate in forest domains and 78 genes in prairie domains. Ninety-seven percent of the prairie lineage-specific genes were located in $P_{mix}$, suggesting that the mixing of prairies into forests does associate with lineage specification during implantation. Meanwhile, the prairie genes that move from compartment B to A during implantation are also functionally enriched in ectoderm differentiation and in utero embryonic development (Figure S5C), in accordance with the above analysis on $P_{mix}$ regions.

## 4. Discussion

### 4.1. Sequence-Based Chromatin Domain Segregation 

In the present study, we described the chromatin structural changes during embryonic development from the zygote to the post-implantation stages and, in particular, how the DNA sequence is associated with the chromatin structural changes. We also investigated the relationship between DNA domain segregation and genetic/epigenetic properties. Earlier studies have shown a gradual compartmentalization in embryonic development [4]. Here, we connect the 3D structure change to DNA sequence properties and perform a functional analysis of these structural changes. The sequence-structure-function analysis provides a common ground for the understanding of different biological processes. 

In particular, two types of genomic domains as defined earlier, forests and prairies, are shown to generally undergo an enhanced spatial separation in early embryonic development in both mouse and human embryos, which is also reflected in the DNA methylation and gene expression difference between the two domain types. In fact, it is possible that the different behaviors of DNA with different sequence properties could be a result of the different epigenetic events and different proteins differentially enriched at forest/prairie domains, which are DNA-sequence dependent. It was reported that L1 and B1 became gradually segregated during early embryonic development [43]. Herein, we divided the human genome into four parts: high-CpG-high-SINE (hChS), high-CpG-low-SINE (hClS), low-CpG-high-SINE (lChS) and low-CpG-low-SINE (lClS) (see “Methods”) and compared the segregation behaviors among hChS and hClS, lChS and lClS, hChS and lChS, hClS and lClS (see “Methods”), aiming to identify the fundamental sequence factors in chromatin segregation. The results revealed that the segregation extent of the latter two groups (that is, hChS and lChS (1.238 and 1.115), hClS and lClS (1.257 and 1.224)) was generally higher than the former groups (hChS and hClS (1.164 and 0.939), lChS and lClS (1.127 and 0.983)), indicating that compared to interspersed elements, CpG density variation may play a fundamental role in chromatin compartmentalization. 

Interestingly, noticeable domain mixing does occur at two important stages, ZGA, when zygotic genes begin to express, and implantation, during which differentiation starts to form germ layers. During the mouse ZGA process, short-range (<500 kb) forest–prairie spatial interactions increase, which event was thought to be associated with gene activation since our previous work has revealed that prairie genes tend to move to a more forest environment for activation [29]. Besides, we also investigated in detail the chromatin structural changes during the ZGA process at different scales. Notably, from human 2-cell to 8-cell, we did not observe domain mixing (Figure 1B). It is believed that human ZGA begins at ~4-8-cell stage; unfortunately, the Hi-C data of the human 4-cell is not available. Structural analyses between the 2-cell and 4-cell, as well as between 4-cell and 8-cell, may help us to gain more insights into the relationship between the human ZGA and chromatin structure. During implantation, we observed a conspicuous decrease in the segregation ratio $R_{s}\left(d\right)$ for prairies at large genomic distances (>3 Mb). Intriguingly, almost all mouse E6.5 epiblast-specific prairie genes resided in $P_{mix}$ domains, again emphasizing the intimate link between domain mixing and gene activation (lineage specification). Genes in the prairie domains that become more mixed in implantation are prominently associated with ectoderm differentiation.

**Figure 6:**
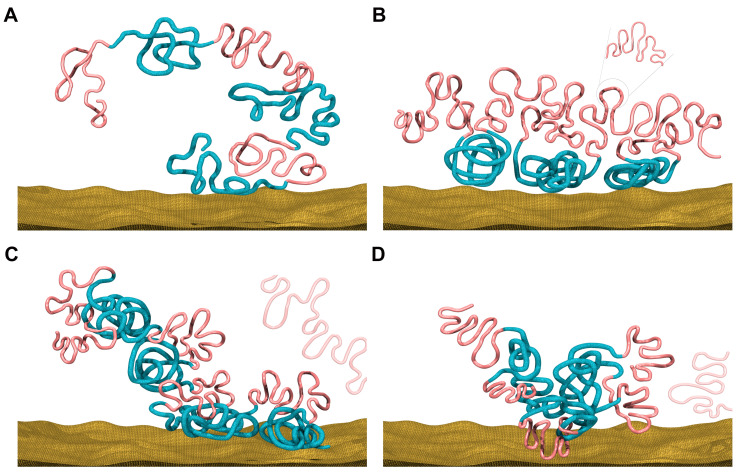
Schematic pictures of chromatin structural patterns at different stages. (**A**) In the beginning zygotic stage, chromatin has few long-range structural features. Here, the red lines represent CGI forest domains, and the blue ones represent CGI prairie domains. (**B**) In ICM stage, the two different types of domains segregate from each other to form compartments. (**C**) In differentiated cells, prairies containing tissue-specific genes tend to form contacts with forests. (**D**) During senescence, prairies congregate further (with an increased probability to detach from the nuclear membrane).

An analysis of Hi-C data in differentiated and senescent cells [44] showed a consistently enhanced segregation of chromatin structures, with a gradual establishment of long-range DNA contacts from zygote to senescence. At the zygotic stage, few high-order structural features exist, and the chromatin is organized similarly to a random coil (Figure 6A). As the embryo develops, local structures (loops and TADs) become more prominent and the two different types of domains segregate from each other to form compartments. Such a trend continues through ICM (Figure 6B). After implantation and as differentiation starts, a subset of prairie domains tends to mix into the active environment, activating associated genes (Figure 6C). During senescence, prairie domains congregate further, some of which detach from nuclear membranes and cluster inside the nucleus, permitting long-range contact establishment [45] (Figure 6D). Our previous analysis showed that chromatin domain segregation continues in cell differentiation and senescence [44]. It appears that the overall trend of chromatin structure change follows the establishment of long-range contacts from birth to senescence, as seen from the segregation level change (Figure S6A–C). In this process, intra-domain contacts are established first, followed by long-range inter-domain contact formation, and then the establishment of lineage-specific inter-domain contacts, which relates to cell differentiation (Figure S6D–G).

### 4.2. The Association between Transcription Inhibition and Genome Architecture

In this study, we also investigated the association between transcription and chromatin structure. The increase in the F–P ratio at small genomic distances (<500 kb) tends to be associated with ZGA. The effect of transcription on domain mixing during ZGA can also be tested using available mouse α-amanitin-treated Hi-C data. To investigate how transcription inhibition affects the chromatin architecture, we compared the F–P and P–P ratios within the top 10% contact probabilities under one certain genomic distance between the PN3, normal late 2-cell, and α-amanitin-treated-20 h cell (α-amanitin-treated late 2-cell). The critical genomic distance of α-amanitin-treated-20 h cell is significantly smaller than that of the normal late 2-cell, and the F–P and P–P ratios of α-amanitin-treated-20 h cell are between PN3 and normal late 2-cell (Figure S7A), indicating that transcription inhibition can slow down the establishment of long-range contact and forest–prairie separation. We further compared the F–P and P–P ratios between the α-amanitin-treated-20 h cell and α-amanitin-treated-45 h cell, and found that the critical genomic distance and P–P ratio of the latter are larger than the former, the F–P ratio of the latter is smaller than the former (Figure S7B), hinting that when transcription is inhibited, long-range chromatin contacts can still be established, and forest–prairie separation also progresses, although at a reduced rate. This observation is in keeping with previous findings that the maturation of higher-order chromatin organization can at least partially proceed in the absence of zygotic transcription [4].

### 4.3. Other Factors That May Relate to Chromatin Structure Change

Sequence-based chromatin-domain segregation suggests that factors affecting domain interactions could lead to functional chromatin structure changes. The cell cycle is an important factor that could influence chromatin domain segregation. During embryonic development, the establishment of a TAD structure was reported as requiring DNA replication but not zygotic transcription [4,5], implying the critical role of cell division in 3D structural formation. In fact, our analysis shows that the different cell division behaviors of different tissues, including those derived from different germ layers, further increase their discrepancy in structural segregation as well as in methylation pattern. The current and earlier analyses indicate that DNA methylation is in fact associated closely with chromatin conformation [21]. The age-related hypomethylation appears to closely track the accumulation of cell divisions [36]. We speculate that, along with hypomethylation (leading to the formation of partially methylated domains), prairie domains tend to segregate more stably in the less active spatial domains of heterochromatin [46].

During mouse embryonic development (from zygote to blastocyst), the nucleocytoplasmic ratio gradually increases, accompanied by a decrease in nuclear size [47]. The change of nucleocytoplasmic ratio, in principle, could have an effect on the decay of contact probability by affecting the crosslinking efficiency in the Hi-C experiment, although in the procedure of sisHi-C, excessive reagent was used to ensure a sufficient and quick fixation. In addition, nuclear lamina is known to play an indispensable role in chromatin structure formation and maintenance. LADs contribute to priming B compartments and remain as the most stable chromatin domains, playing an important role in global chromatin segregation. One previous study [48] also unveiled a connection between nuclear size and nuclear lamina, which may also contribute to a change to the landscape of chromatin domain segregation. These issues remain to be fully addressed by experimental studies.

Since precise gene expression profiles are essential for normal embryo development, factors influencing gene regulation were suggested as impacting the development process [49]. For instance, OCT4, one of the four Yamanaka factors, was thought to play a vital role in development. Accurate epigenetic patterns (such as DNA methylation, histone modifications), as well as high order genome organization, are needed to ensure the normal regulation of OCT4. Therefore, it is worthwhile to understand the regulatory mechanism behind specific gene regulation during development. These include but are not limited to how DNA methylation, active/repressive histone mark loading, and chromatin domain segregation vary before and after gene activation/repression. It is interesting to uncover the interplay between different epigenetic makers and the high-order chromatin structure, to gain more insight into the roles that these various factors play in embryo development.

## 5. Conclusions

In this study, we delineated chromatin structural and epigenetic reprogramming in early embryonic development, based on the sequence-based domain segregation model, and correlated the overall chromatin structural changes with functional implementation during ZGA and implantation. The two types of DNA domains gradually separate from each other during embryonic development, but they show a tendency to mix when transcription and implantation happen. Increased interactions between the two types of domains indicate the start of transcription or set the stage for further cell differentiation, leading to lineage commitment. Genes of changed segregation states show lineage-relevant functions and important roles in the corresponding processes; thus, more detailed analyses on gene expression and the regulation of transcription factors are needed for understanding the related molecular mechanisms and, more importantly, to predict biological functions at the molecular level from the perspective of domain segregation and mixing-related chromatin structure formation.

## Data Availability

All data analyzed during this study are publicly available. The detailed data accession can be found in Additional file 2: Data_Sources.xlsx.

## Supplementary Materials

The following are available online at https://www.mdpi.com/article/10.3390/cells10102521/s1, Additional File 1: Supplemental information.docx (Supplementary text: Further investigation on the relationship among sequence, compartmentalization and expression, Figure S1: Domain segregation in early embryonic development, Figure S2: Comparisons between maternal and paternal genomes, Figure S3: Compartment changes related to domain segregation, Figure S4: Domain segregation and differentiated DNA methylation associated with germ layer formation, Figure S5: Implantation-related domain mixing in differentiation, Figure S6: Chromatin structural patterns at different stages, Figure S7: The association between transcription inhibition and genome architecture), Additional file 2: Data_Sources.xlsx.
